# Lifecourse Social Position and D-Dimer; Findings from the 1958 British Birth Cohort

**DOI:** 10.1371/journal.pone.0093277

**Published:** 2014-05-08

**Authors:** Faiza Tabassum, Meena Kumari, Ann Rumley, Chris Power, David P. Strachan, Gordon Lowe

**Affiliations:** 1 Third Sector Research Centre, Faculty of Human and Social Sciences, University of Southampton, Southampton, United Kingdom; 2 Department of Infection and Population Health, University College London (UCL), London, United Kingdom; 3 Department of Epidemiology and Public Health, University College London (UCL), London, United Kingdom; 4 ISER, Essex University, Wivenhoe Park, Colchester, United Kingdom; 5 Institute of Cardiovascular and Medical Sciences, University of Glasgow, Royal Infirmary, Glasgow, United Kingdom; 6 Centre for Pediatric Epidemiology and Biostatistics, Institute of Child Health, University College London (UCL), London, United Kingdom; 7 Division of Population Health Sciences and Education, St George's, University of London, London, United Kingdom; Hunter College, City University of New York (CUNY), CUNY School of Public Health, United States of America

## Abstract

The aim is to examine the association of lifecourse socioeconomic position (SEP) on circulating levels of D-dimer. Data from the 1958 British birth cohort were used, social class was determined at three stages of respondents' life: at birth, at 23 and at 42 years. A cumulative indicator score of SEP (CIS) was calculated ranging from 0 (always in the highest social class) to 9 (always in the lowest social class). In men and women, associations were observed between CIS and D-dimer (*P*<0.05). Thus, the respondents in more disadvantaged social classes had elevated levels of D-dimer compared to respondents in less disadvantaged social class. In multivariate analyses, the association of disadvantaged social position with D-dimer was largely explained by fibrinogen, C-reactive protein and von Willebrand Factor in women, and additionally by smoking, alcohol consumption and physical activity in men. Socioeconomic circumstances across the lifecourse at various stages also contribute independently to raised levels of D-dimer in middle age in women only. Risk exposure related to SEP accumulates across life and contributes to raised levels of D-dimer. The association of haemostatic markers and social differences in health may be mediated by inflammatory and other markers.

## Introduction

Fibrin D-dimer, the most commonly used clinical assay of coagulation activation and in vivo fibrin formation and lysis in circulating blood has been associated with increased risk of cardiovascular disease. A meta-analysis of prospective studies [1] showed an association of circulating levels of fibrin D-dimer with coronary heart disease (CHD) that appeared of similar strength to that of fibrinogen (a biochemical marker of an existing thrombophilia, which is correlated in part due to associations with asymptomatic and symptomatic arterial lesions [2]). While it has been suggested that D-dimer and other fibrin(ogen) degradation products may have a pro-inflammatory effect [2], the association of D-dimer and CHD risk appears independent of inflammatory markers, such as fibrinogen, C-reactive Protein (CRP) and interleukin-6 [3], [4]. Additionally, D-dimer appears to be a consistent, independent predictor of recurrent venous thromboembolism (VTE) [5]–[7]. Whether or not associations between D-dimer and cardiovascular risk are independent of other vascular risk factors is controversial. Hemostatic factors proved to be directly and independently correlated with the risk of subsequent coronary events [8]–[11]. Elevated D-dimer values occur in various disorders in which the coagulation system is excessively activated, such as acute venous thromboembolism [6], [7]. High plasma levels of D-dimer have been associated with the incidence of cardiovascular disease [1], [2], [8]–[13], and vascular dementia [14], [15].

Recent studies showed that D-dimer and hemostatic and inflammatory markers such as von Willebrand factor (vWF; an endothelial marker), CRP and fibrinogen are positively associated with atherosclerosis and CHD [2]. Hemostatic markers namely vWF and tissue plasminogen activator antigen (t-PA) may be a mechanism linking socioeconomic position (SEP) with (CHD) [16], [17]. We have shown, using data from the 1958 British Birth cohort that vWF and t-PA are influenced by early life circumstances in addition to accumulation of adversity [16]. These data suggest that mid-life measures of hemostatic markers may reflect early life influences. However, this has not been explored in a British population. In a study of elderly males, the most deprived current social class was associated with elevated levels of D-dimer but only in unadjusted models [18], however, this study did not investigate the associations of D-dimer with the lifecourse effects of SEP in middle aged men and women. The impact of cumulative exposure to disadvantage over the lifecourse in addition to time sequencing has been hypothesized to be important to health. The accumulation hypothesis states that adverse factors occur at different stages in life and accumulate over time and critical period model argues that early life influences biological development independently, this is also known as ‘biological programming’ [19]–[22].

We investigated the effects of SEP measured by occupational class at three time points (birth, at 23 and 42 years) throughout the lifecourse on circulating levels of D-dimer measured in adulthood (45years) in a cohort of individuals born in one week in 1958. We examined whether the association of D-dimer with SEP is cumulative, i.e. the increased exposure to adverse SEP is associated with raised levels of D-dimer and whether the associations between the accumulation of SEP and D-dimer were attributable to major risk factors for CHD.

## Methods

### Ethical approval

Ethical approval for the medical examination and consent to obtain biomedical information was obtained by the 1958 British Birth Cohort team from South East MREC (ref: 01/1/44). Written consent was obtained from each participant to take part in the medical examination.

### Data

The 1958 British Birth Cohort is a continuing longitudinal representative survey of predominantly white people (97%) living in Great Britain who were born in one week in 1958. The subsequent surveys were collected when participants were aged 7, 11, 16, 23, 33 and 42 with medical data at 45years. A detailed description of the cohort members at age 45 is provided elsewhere [23], [24]. All cohort members who were still in contact with the cohort study team, and who at age 41–42 had not required a proxy interview were invited to participate in a clinical examination in their homes at the age of 44–45 years. The target sample at age 45 was 11971 people, 75% of the eligible sample of survivors and non-emigrants; 9377 people participated in the medical survey. Despite attrition people remained representative of the original sample, including with respect to blood samples [24]. This study is an analysis of previously collected data and therefore ethical approval was not required for this study.

### Blood collection and measurement

Fibrin D-dimer was assessed from venous blood samples that were obtained without prior fasting and posted to collaborating laboratories [25]. Fibrin D-dimer was measured on stored samples at the end of the field study period by ELISA (Hyphen, Paris, France) and standardized for interbatch variation. All analytes were monitored for internal quality control by Levey-Jennings plots during the assay period. The intra-assay and inter-assay coefficients of variation for fibrinogen, CRP, vWF and D-dimer are acceptable as: 2.6 & 3.7%, 4.7 & 8.3%, 3.3 & 4.2% and 7 & 8% respectively.

### Health behaviors

Data on smoking and physical activity were collected by questionnaire at age 42. The respondents were classified as never smokers, ex-smokers and current smokers. At age 42, participants responded to a single question about the frequency of leisure physical activity. Three levels of physical activity were created: high (respondents who reported ≥4 days/week physical activity), intermediate (respondents who reported 1–3 days/week physical activity) and none/low (respondents who reported no or less frequent physical activity). Alcohol consumption at 42 was recorded in the following categories: drinking most days of the week, 1, 2 or 3 times a week, 1, 2 or 3 times a month, less often and never.

### Cardiovascular risk factors

Measurements of height and weight were recorded during the clinical examination. Body mass index (BMI) was calculated as weight in kilograms divided by the square of height in meters (kg/m^2^). The following cut-offs were used to categorize BMI: up to normal (BMI<25), overweight (BMI 25 or more and less than 30) and obese (BMI 30 or more). Blood pressure was measured using the Omron 705CP automated sphygmomanometer (Omron, Tokyo, Japan). Mean blood pressure was determined for readings that the nurse considered to be reliable. Total cholesterol was measured by autoanalyser. Fibrinogen and high-sensitivity CRP were assayed by immuno-nephelometry (Dade Behring) and vWF antigen was measured by ELISA (DAKO) [25]. The Framingham risk score was calculated [26] based on the values of total cholesterol, HDL cholesterol, systolic and diastolic blood pressure, diabetes, age, gender and smoking status.

### Socioeconomic position

The present report uses occupational class to represent SEP. Registrar General's (RG) social class system which is a measure of social class based on occupation was measured directly from the participants when they were adults and from their parents during their childhood. The social classes are described as: I-professional occupations, II- managerial and technical occupations, IIIN-skilled occupations (non-manual), IIIM- skilled occupations (manual), IV-partly-skilled occupations and V-unskilled occupations. For analysis purposes, we have combined social classes I & II (SC1) and social classes IV & V (SC4) while other classes are social class IIIN; non-manual (SC2) and social class IIIM; manual (SC3). Categories were combined in this way because of small numbers in some categories of social class.

A cumulative indicator score of SEP (CIS) during three occasions (childhood at birth, early adulthood at 23 and middle of life at 42) was constructed by means of a total social class lifecourse score ranging from 0 to 9. Social classes (SC1, SC2, SC3 & SC4) were assigned scores 0, 1, 2 and 3 respectively. These scores were added to create the CIS. Hence, CIS ranges from 0 (0+0+0, i.e., SC1 at all three occasions) to 9 (3+3+3, i.e., SC4 at all three occasions). Likewise, CIS 1 corresponds to (0+1+0, 0+0+1, 1+0+0) through to CIS 8 which corresponds to (2+3+3, 3+2+3, 3+3+2).

### Statistical analysis

Data on D-dimer and social class at birth and at 42 were available for 5937 respondents of which 3130 were men and 2807 were women. The analyses were done separately for men and women because of the variations in D-dimer levels between gender and also social position based on occupation may have a different interpretation for men and women. Normality of D-dimer was assessed, and data were log transformed prior to analyses. Geometric means are presented, and the natural log of the concentrations was used in the regression models. The relationship between D-dimer and social class was explored using ANOVA, equality of variances was tested by Bartlett test, the differences between the most deprived and the highest social classes were tested using the test command in Stata. Regression analysis was used to explicitly test for a trend in the means of the outcome variable across social class by entering social class as a continuous variable in the model. Mutually adjusted means were determined in models with terms present for social class at birth, at 23 and own recent (42 years) social class. Unadjusted and adjusted associations of accumulation of SEP (CIS) with D-dimer were estimated by using simple and multiple regression analysis. The collinearity between variables was checked by the variance inflation factors (VIF) which was under the reasonable limits indicating that collinearity was not an issue. The multivariate analyses included respondents with complete data on all variables (n = 5367).

## Results

### D-dimer and social class

Mean levels of D-dimer were significantly higher (*P*<0.001) in women than in men (mean levels were: 186.8 and 137.0 ng/mL in women and men, respectively). Table 1 shows the mean concentrations of D-dimer with the four categories of social class at three time points. A social class gradient at all three time points was observed for D-dimer in men (*P*≤0.005). Thus, the respondents in more disadvantaged social classes (SC3/SC4) had elevated levels of D-dimer compared to respondents in less disadvantaged social class (SC1). In women, there was a trend for D-dimer by social class at birth and at 42 years, although the trend was borderline for social class at 23 (*P* = 0.071). Bartlett test for equality of variances was non-significant (*P*>0.05) indicating that the use of ANOVA is appropriate.

**Table 1:** Geometric Means and standard deviation of D-dimer (ng/mL) concentration at 45 years by the social class at three occasions in the 1958 British Birth cohort.

	SC at birth			SC at 23			Recent SC (42years)		
	No.	Mean	SD	No.	Mean	SD	No.	Mean	SD
Men (n = 3130)									
SC1	655	131.37	1.75	867	130.32	1.72	1531	131.63	1.73
SC2	321	137.69	1.80	550	135.64	1.84	290	138.38	1.88
SC3	1541	137.14	1.72	1207	139.77	1.72	990	141.17	1.72
SC4	613	140.89	1.77	506	142.59	1.74	319	142.59	1.73
*P*_trend_	0.032			<0.001			0.001		
Women (n = 2807)									
SC1	542	181.27	1.75	718	179.47	1.68	113	181.27	1.68
SC2	282	169.02	1.62	1363	188.67	1.73	949	184.93	1.73
SC3	1431	188.67	1.70	264	194.42	1.65	199	185.86	1.67
SC4	552	192.48	1.68	462	188.67	1.63	546	198.34	1.66
*P*_trend_	0.012			0.071			0.004		

SC indicates social class; SC1, professional & managerial; SC2, non-manual; SC3, manual; SC4, unskilled.

SD, standard deviation.

Table 2 shows the results of the regression analyses with social class at three occasions fitted individually and simultaneously. In men, social class at birth, at 23 and at 42 was not associated with D-dimer in mutually adjusted analysis, however the test of joint significance was significant (p = 0.001) indicating the collective contribution of social class at three occasions on levels of D-dimer is significant. In women, social class at 23 was not associated with D-dimer in the simultaneously adjusted analyses but social class at birth and at 42 remained associated in the adjusted analysis also the joint significance test was significant (p = 0.005).

**Table 2:** The unadjusted and simultaneously adjusted regression analyses of D-dimer at 45years with social class at three stages of life in the 1958 British birth cohort.

	SC at birth		SC at 23		Recent SC (42years)	
	β*	95% CI	β*	95% CI	β*	95% CI
**Men** (n = 3130)						
Unadjusted	0.02	0.00–0.04	0.03	0.01–0.05	0.03	0.01–0.05
*p*_for trend_	0.032		<0.001		0.001	
Adjusted ‡	0.01	−0.01–0.03	0.02	−0.00–0.04	0.02	−0.00–0.04
*p* for trend	0.36		0.07		0.10	
Joint test of sig†	0.001					
**Women** (n = 2808)						
Unadjusted	0.03	0.01–0.05	0.02	−0.00–0.04	0.03	0.01–0.04
*p*_for trend_	0.012		0.07		0.004	
Adjusted ‡	0.02	−0.00–0.04	0.00	−0.02–0.03	0.02	0.00–0.04
*p*_for trend_	0.05		0.67		0.03	
Joint test of sig†	0.005					

SC, social class; CI, confidence interval.

* Log transformed regression coefficients (β).

‡ Simultaneously adjusted with social class at three occasions.

† Joint test of significance of social class at three stages of life

### D-dimer and risk factors for cardiovascular disease

The association of D-dimer with potential risk factors was examined (Table S1). In men, current and ex-smokers had raised levels of D-dimer compared with never smokers (*P* = 0.05). In women this association was not significant. Levels of physical activity were not associated with D-dimer levels. Alcohol consumption was negatively associated with levels of D-dimer. D-dimer levels were lowest among normal weight people compared to overweight and obese. A gradient in D-dimer levels across the tertiles of Framingham score, fibrinogen, CRP and vWF were observed for men and women (*P*<0.001) such that mean D-dimer levels were higher for those in the medium and highest tertile compared to the lowest tertile. Positive correlations were observed between D-dimer and CRP (0.14), fibrinogen (0.18) and vWF (0.18).

Analyses examining each risk factor and CIS score (table 3) indicate that those belonging to CIS 0 (always been in the highest social class) had the lowest mean Framingham score, fibrinogen, CRP and vWF levels, low prevalence of current smoking, low rates of physical inactivity, higher consumption of alcohol and low prevalence of obesity compared to those with higher CIS scores (*P*<0.01).

**Table 3:** The associations between the risk factors and cumulative indicator score of SEP (CIS) in the 1958 British birth cohort. Values are mean or prevalence.

Risk factor	Cumulative Indicator Score of SEP (CIS)											
	0	1	2	3	4	5	6	7	8	9	*P*_for trend_	Total sample
**Men**												
Mean Framingham score	5.5	5.7	5.8	5.9	6.1	6.2	6.3	6.5	6.6	6.7	<0.01	6.1
95% CI	5.4–5.7	5.6–5.8	5.7–5.9	5.9–6.0	6.0–6.2	6.1–6.3	6.2–6.4	6.4–6.6	6.5–6.8	6.6–6.9		6.0–6.2
Mean Fibrinogen (g/L)	2.7	2.8	2.8	2.8	2.9	2.9	2.9	2.9	2.9	3.0	<0.01	2.85
95% CI	2.7–2.8	2.7–2.8	2.8–2.8	2.8–2.8	2.8–2.9	2.8–2.9	2.8–2.9	2.9–3.0	2.9–3.0	2.9–3.0		2.8–2.9
Mean CRP (mg/L)*	0.7	0.8	0.8	0.9	0.9	1.0	1.0	1.1	1.1	1.2	<0.01	0.9
95% CI	0.7–0.8	0.7–0.8	0.8–0.9	0.8–0.9	0.9–1.0	0.9–1.0	1.0–1.01	1.0–1.2	1.1–1.2	1.1–1.3		0.9–1.0
Mean vWF (IU/dL)	118	119	120	121	122	123	124	125	126	127	<0.01	122
95% CI	115–121	116–121	118–122	119–123	121–124	122–125	122–126	123–127	123–129	124–130		121–124
Current smoker (%)	10	12	13	13	15	21	30	34	39	40	<0.01	21
95% CI (%)	6–13	8–17	10–17	10–17	12–19	16–27	25–33	29–39	31–47	25–55		19–22
Physical inactivity (%)	15	12	18	16	21	26	27	31	30	38	<0.01	22
95% CI (%)	10–19	7–16	14–21	12–20	17–25	21–32	23–31	27–36	22–37	22–53		21–24
Alcohol cons (%)†	36	37	34	27	26	19	19	20	20	15	<0.01	26
95% CI (%)	30–42	30–44	29–39	22–32	21–30	14–24	15–22	16–24	24–27	4–26		24–27
Obese‡ (%)	15	15	19	22	30	28	28	30	32	28		0.25
95% CI (%)	11–19	10–19	15–23	18–26	26–35	22–33	24–32	25–34	25–39	13–42	<0.01	23–26
**Women**												
Mean Framingham score	3.1	3.3	3.6	3.8	4.1	4.4	4.6	4.9	5.1	5.4		4.1
95% CI	2.8–3.3	3.1–3.5	3.4–3.8	3.7–4.0	4.0–4.2	4.2–4.5	4.4–4.8	4.7–5.1	4.9–5.4	5.1–5.6	<0.01	3.9–4.2
Mean Fibrinogen (g/L)	2.8	2.9	2.9	2.9	3.0	3.0	3.1	3.1	3.1	3.2	<0.01	3.0
95% CI	2.8–2.9	2.8–2.9	2.9–2.9	2.9–3.0	2.9–3.0	3.0–3.1	3.0–3.1	3.0–3.1	3.1–3.2	3.1–3.2		2.9–3.0
Mean CRP (mg/L)*	0.7	0.8	0.8	0.9	1.0	1.1	1.2	1.3	1.4	1.5	<0.01	1.0
95% CI	0.6–0.7	0.7–0.8	0.8–0.9	0.8–1.0	0.9–1.0	1.0–0.1	1.1–1.2	1.2–1.4	1.2–1.5	1.3–1.7		0.9–1.0
Mean vWF (IU/dL)	118	119	119	120	120	121	121	122	122	123	0.16	120
95% CI	115–121	116–121	117–121	118–121	119–122	119–122	119–123	119–124	119–125	119–127		119–122
Current smoker (%)	8	11	16	19	18	27	29	42	38	53	<0.01	22
95% CI (%)	4–13	6–16	12–19	15–23	15–21	22–32	24–34	35–50	29–46	39–66		21–24
Physical inactivity (%)	20	16	23	25	26	24	31	39	38	43	<0.01	27
95% CI (%)	14–26	10–22	19–27	20–29	22–30	19–28	26–37	32–46	29–46	30–57		25–28
Alcohol cons (%)†	25	24	23	18	14	13	12	8	7	4	<0.01	16
95% CI (%)	19–32	17–31	19–27	14–22	11–17	9–17	9–16	4–12	3–12	−1–9		15–18
Obese‡ (%)	14	16	22	23	19	22	26	30	29	24	<0.01	22
95% CI (%)	8–19	10–22	18–26	19–28	16–22	18–27	21–31	23–37	21–37	13–36		20–24

CI, confidence interval.

*Geometric means presented.

† High alcohol consumption: alcohol consumption on most of the days

‡ Obese: BMI ≥ 30.

### Multivariate analyses of D-dimer with social class

Unadjusted (Model 1) and risk factor adjusted associations of D-dimer with CIS are shown in Table 4. CIS was associated with logarithmic concentrations of D-dimer in both sexes (men: β = 0.016, *P*<0.001) and (women: β = 0.014, *P* = 0.002) (regression coefficient β represents the change in logarithmic concentration of D-dimer per one score change in CIS.

**Table 4:** Change in the regression coefficient of Cumulative Indicator Score of SEP (CIS) in association with D-dimer without- and with-adjustments with risk factors in the 1958 British Birth Cohort.

Models	Men (n = 2832)			Women (n = 2535)		
	*β* coefficient*	% change in *β* coefficient†	*P*_trend_	*β* coefficient	% change in *β* coefficient	*P*_trend_
1	0.016		<0.001	0.014		0.002
2	0.011	31.25%	0.01	0.010	28.57%	0.04
3	0.007	56.25%	0.07	0.003	78.57%	0.53
4	0.012	25%	0.004	0.008	43%	0.09
5	0.006	63%	0.16	0.001	92.86%	0.82

Model 1: Association between D-dimer and Cumulative Indicator Score of SEP (CIS).

Model 2: Model 1 plus smoking, physical activity and alcohol consumption.

Model 3: Model 1 plus fibrinogen, C-reactive protein and vWF.

Model 4: Model 1 plus Framingham risk score and BMI.

Model 5: Model 1 plus all the factors mentioned above.

* Each unstandardised regression coefficient (*β*) represents the amount of change in the logarithmic concentration of D-dimer for an increase in CIS.

† This is the change in the regression coefficient of CIS in the regression models compared to Model 1 e.g., ((*β*_Model 1_ − *β*_Model 2_)/*β*_Model 1_)*10.

In Model 2 (Model 1 + smoking, physical activity and alcohol intake), the association of CIS with D-dimer was robust to further adjustment for health behaviors (*P*<0.05). In men and women, the associations between CIS and D-dimer became non-significant when adjusted with fibrinogen, CRP and vWF (Model 3). These associations were similar with and without inclusion of fibrinogen. The associations between D-dimer and CIS are shown in Model 4 following adjustment with Framingham risk score and BMI; in men D-dimer remained significantly associated with CIS while in women it became non-significant. The associations between D-dimer and CIS became non-significant when adjusted with all the factors (Model 5: health behaviors, inflammatory markers and cardiovascular risk factors).

Health behaviors explained 31% of the gradient in men and 29% in women while inflammatory markers (fibrinogen, CRP and vWF) explained 56% and 79% respectively in men and women and abolished the association between D-dimer and CIS. Framingham score and BMI explained 25% of the gradient in men and 43% in women (non-significant trend). When all the above mentioned factors were adjusted together they explained 63% of the gradient in men and 93% in women to a non-significant trend. Obtained from the final model (Model 5), the proportion of variance of log D-dimer explained by socioeconomic position (CIS) was 8% in men and 13% in women. A sensitivity analysis was also conducted by excluding those with heart or kidney diseases from our analyses (10% of the total sample) and the results remained unchanged.

Respondents with a high score of CIS had higher mean levels of D-dimer compared with those with a score 0. When adjusted with additional risk factors the pattern becomes weaker and non-significant (Table S2).

## Discussion

Our results indicate that there are social differences in the level of D-dimer, such that increased exposure to adverse SEP across the lifecourse is associated with elevated levels of D-dimer in the mid- life. However, these associations were largely, explained by CRP, fibrinogen and vWF and other traditional CHD risk factors. We found that socioeconomic circumstances across the lifecourse also contribute independently to raised levels of D-dimer in middle age among women but not in men.

The magnitude of the difference in D-dimer levels between the most advantaged and most disadvantaged groups was substantial and accord with a potential role for D-dimer in the pathways that mediate increased cardiovascular risk in disadvantaged social groups. One possible explanation might be that as D-dimer reflects fibrinogen levels as it is a measure of fibrin turnover and it has been shown that fibrinogen levels were raised among those in lower concurrently assessed measures of social position [27]–[29]. We are in line with other studies [2] that report the positive and significant correlations between D-dimer and fibrinogen. However, our findings are not driven by this correlation suggesting that inflammatory processes account for much the pro-coagulant state in those with disadvantaged social position assuming there are no measurement issues. Additionally we observe positive associations with CRP fibrinogen and vWF which supports our hypothesis that there may be a link between these respective markers of inflammation and D-dimer in middle-aged men and women.

A recent study has shown that high levels of education and occupation tended to be associated with lower risks for VTE [30]. In another study both persistent stress and low occupational class were independently related to future pulmonary embolism: the mechanisms are unknown, but effects on coagulation and fibrinolytic factors are likely [31]. Our report shows the lack of step-wise gradient in CIS in addition to the fact that mostly, D-dimer did not survive full mutual adjustment with SEP at three time points indicating that the effects of accumulation and critical periods are interrelated in our report particularly among women which is in line with the findings reported by Hallqvist et al. [21] and Tabassum et al. [16].

D-dimer levels are associated with smoking, physical inactivity, alcohol consumption and all other risk factors used in the analyses. Our results following adjustment for these measures show a reduction in the strength of D-dimer with the accumulation of social class, which argues for health behaviors and the CHD risk factors used in this study as partial mediators of social differences in CHD. Our study suggests that disadvantaged social position is accompanied by a pro-coagulant and pro-inflammatory profile that is distinct from a metabolic profile described in other studies to be associated with social disadvantage [27]. This pro-coagulant and pro-inflammatory profile appeared to be related to some health behaviors (smoking, physical activity and alcohol consumption) in men [32], [33]. It is possible that sub-clinical ill health is mediating some of the relationship between social position and D-dimer. However, the failure of metabolic markers and measures of health such as hypertension to explain the social gradient in D-dimer suggests that these health behaviors do not contribute.

The strengths and limitations of our study need to be considered. First, the findings were based on a large scale national level survey in Britain. Secondly, D-dimer was measured in a large cohort of similar age and ethnicity. Other limitations to this study which need to be considered. First, not all the respondents who were part of the perinatal survey in 1958 were seen at follow-up. However, previous work reports that biases between respondents and non-respondents are negligible in this cohort [24]. We did not have other measures of CHD status to control the associations of D-dimer with SEP as well as we cannot adjust for underlying co-morbidities, for example atherosclerosis. However, sensitivity analyses indicate that comorbidities such as heart or kidney diseases do not affect the associations between D-dimer and SEP in our sample. Additionally, we did not have repeated measures of D-dimer and therefore could not examine change in D-dimer with the change in SEP. Any future research on this topic should address the associations of D-dimer levels with social mobility. Finally, we did not address the issue of reverse causation, i.e. the possibility that poor sub-clinical health caused lower social class.

## Conclusions

In conclusion, there is an association of D-dimer and social position such that disadvantaged social class across the lifecourse is associated with raised levels of D-dimer. In multivariate analyses, the association of disadvantaged social position with D-dimer was largely explained by C-reactive protein and von Willebrand Factor in women, and additionally by smoking, physical activity and alcohol consumption in men. The association of haemostatic markers with social class may be mediated by inflammatory and other markers.

## Supporting Information

Table S1 **Associations of risk factors with D-dimer, geometric means (ng/mL) and standard deviations are reported in the 1958 British Birth Cohort.**
(DOCX)

Table S2 **Unadjusted and adjusted geometric means of D-dimer levels (ng/mL) at 45 years by the cumulative indicator score of SEP (CIS) in men and women in the 1958 British birth cohort.**
(DOCX)
