# Supplementary material for: Lifecourse Social Position and D-Dimer; Findings from the 1958 British Birth Cohort
Source: PLoS One. 2014 May 8;9(5):e93277. doi: 10.1371/journal.pone.0093277 (PMC4014469; doi:10.1371/journal.pone.0093277)
Supplement: Table S1 — Associations of risk factors with D-dimer, geometric means (ng/mL) and standard deviations are reported in the 1958 British Birth Cohort. (DOCX) [file pone.0093277.s001.docx]

**Table_S1**

**Supplementary Table 1:** Associations of risk factors with D-dimer, geometric means (ng/mL) and standard deviations are reported in the 1958 British Birth Cohort

|  | Men (n= 2832) | | Women (n= 2535) | |
| --- | --- | --- | --- | --- |
|  | No. | Mean (SD) | No. | Mean (SD) |
| Smoking |  |  |  |  |
| Never | 1346 | 132.95 (1.77) | 1204 | 184.93 (1.70) |
| Ex/occasional | 896 | 130.32 (1.73) | 764 | 179.47 (1.70) |
| Current | 590 | 149.90 (1.66) | 567 | 186.79 (1.63) |
| *P*_trend_ | 0.05 | | 0.851 | |
|  |  |  |  |  |
| Physical activity |  |  |  |  |
| Frequent | 689 | 137. (1.77) | 691 | 181.27 (1.68) |
| Medium | 1515 | 134.29 (1.75) | 1172 | 181.27 (1.68) |
| Low/mild | 628 | 138.38 (1.66) | 672 | 188.67 (1.68) |
| *p*_trend_ | 0.527 | | 0.278 | |
|  |  | |  | |
| Alcohol consumption |  | |  | |
| Most days | 732 | 131.85 (1.75) | 410 | 168.49 (1.70) |
| 1,2,or 3 times a week | 1600 | 134.17 (1.72) | 1315 | 180.06 (1.68) |
| 1,2,or 3 times a month | 252 | 144.95 (1.74) | 296 | 201.76 (1.65) |
| Less often | 184 | 149.36 (1.79) | 393 | 197.12 (1.68) |
| Never | 64 | 143.57 (1.67) | 121 | 200.23 (1.71) |
| *p*_trend_ | 0.001 | | ≤ 0.001 | |
|  |  |  |  |  |
| Body mass index (BMI) |  |  |  |  |
| Normal | 746 | 122.54 (1.74) | 1199 | 165.58 (1.77) |
| Overweight | 1516 | 137.96 (1.71) | 911 | 189.62 (1.66) |
| obese | 741 | 148.06 (1.75) | 596 | 227.08 (1.61) |
| *p*_trend_ |  | ≤ 0.001 |  | ≤ 0.001 |
|  |  |  |  |  |
| Tertiles of Framingham score |  |  |  |  |
| Lowest | 661 | 126.51 (1.75) | 1542 | 175.41 (1.69) |
| Middle | 973 | 132.62 (1.77) | 438 | 188.43 (1.69) |
| Highest | 1198 | 143.55 (1.70) | 555 | 206.26 (1.63) |
| *p*_trend_ | ≤ 0.001 | | ≤ 0.00 | |
|  |  |  |  |  |
| Tertiles of fibrinogen |  |  |  |  |
| Lowest | 1082 | 120.30 (1.80) | 728 | 154.47 (1.73) |
| Middle | 958 | 135.64 (1.65) | 825 | 175.91 (1.65) |
| Highest | 792 | 160.77 (1.66) | 982 | 217.02 (1.60) |
| *p*_trend_ | <0.001 | | <0.001 | |
|  |  |  |  |  |
| Tertiles of CRP |  |  |  |  |
| Lowest | 907 | 121.51 (1.82) | 883 | 160.77 (1.67) |
| Middle | 1029 | 131.63 (1.67) | 761 | 177.68 (1.63) |
| Highest | 896 | 157.59 (1.67) | 891 | 217.02 (1.66) |
| *p*_trend_ | <0.001 | | <0.001 | |
|  |  |  |  |  |
| Tertiles of vWF |  |  |  |  |
| Lowest | 926 | 122.73 (1.72) | 897 | 165.67 (1.68) |
| Middle | 937 | 135.64 (1.70) | 851 | 183.09 (1.63) |
| Highest | 969 | 149.90 (1.75) | 787 | 208.51 (1.70) |
| *p*_trend_ | <0.001 | | <0.001 | |

BMI: normal: bmi<25; overweight: bmi ≥ 25 and obese: bmi ≥ 30
