# Supplementary material for: Lifecourse Social Position and D-Dimer; Findings from the 1958 British Birth Cohort
Source: PLoS One. 2014 May 8;9(5):e93277. doi: 10.1371/journal.pone.0093277 (PMC4014469; doi:10.1371/journal.pone.0093277)
Supplement: Table S2 — Unadjusted and adjusted geometric means of D-dimer levels (ng/mL) at 45 years by the cumulative indicator score of SEP (CIS) in men and women in the 1958 British birth cohort. (DOCX) [file pone.0093277.s002.docx]

Table_S2

Supplementary Table 2. Unadjusted and adjusted geometric means of D-dimer levels (ng/mL) at 45years by the cumulative indicator score of SEP (CIS) in men and women in the 1958 British birth cohort

| CIS | Men | | | | | Women | | | | |
| --- | --- | --- | --- | --- | --- | --- | --- | --- | --- | --- |
|  | n | Model 1* | | Final model† | | n | Model 1* | | Final model† | |
|  |  | Mean | SD | Mean | SD |  | Mean | SD | Mean | SD |
| 0 | 261 | 124.56 | 1.70 | 131.47 | 1.02 | 178 | 172.88 | 1.75 | 182.39 | 1.02 |
| 1 | 186 | 125.56 | 1.79 | 132.49 | 1.02 | 142 | 164.86 | 1.69 | 182.95 | 1.01 |
| 2 | 395 | 134.46 | 1.78 | 133.51 | 1.01 | 391 | 180.68 | 1.70 | 183.52 | 1.01 |
| 3 | 320 | 132.80 | 1.75 | 134.55 | 1.01 | 362 | 188.29 | 1.70 | 184.09 | 1.01 |
| 4 | 410 | 138.71 | 1.68 | 135.59 | 1.01 | 521 | 186.20 | 1.72 | 184.66 | 1.01 |
| 5 | 242 | 132.61 | 1.79 | 136.64 | 1.01 | 301 | 179.78 | 1.62 | 185.23 | 1.01 |
| 6 | 476 | 141.65 | 1.72 | 137.70 | 1.01 | 290 | 190.21 | 1.66 | 185.80 | 1.02 |
| 7 | 353 | 139.66 | 1.71 | 138.76 | 1.02 | 172 | 189.60 | 1.64 | 186.38 | 1.02 |
| 8 | 149 | 146.01 | 1.73 | 139.84 | 1.02 | 125 | 191.57 | 1.56 | 186.95 | 1.03 |
| 9 | 40 | 139.26 | 1.72 | 140.92 | 1.02 | 53 | 207.82 | 1.80 | 182.39 | 1.02 |
| *P* _for trend_ |  | 0.016 | | 0.11 | |  | 0.014 | | 0.78 | |

CIS: Cumulative Indicator Score of SEP

*Model 1: Association between D-dimer and CIS.

†Final model: Adjusted for smoking, physical activity, alcohol consumption, fibrinogen, CRP, vWF, Framingham score and BMI.
